# Correlations of Post-stroke Depression with Inflammatory Response Factors

**Published:** 2018-07

**Authors:** Yuyuan MU, Zhichao WANG, Jiexin ZHOU, Chenxi TAN, Hongjiao WANG

**Affiliations:** 1. Dept. of Neurology, Second Affiliated Hospital of Qiqihar Medical College, Qiqihar Heilongjiang, 161000, P.R. China; 2. Dept. of Academic Research, Qiqihar Medical University, Qiqihar, 161000, P.R. China

**Keywords:** PSD, IL-6, TNF-α, CRP, Correlation

## Abstract

**Background:**

We aimed to investigate the correlations of the inflammatory response factors, interleukin-6 (IL-6), tumor necrosis factor-α (TNF-α), high-sensitivity C-reactive protein (CRP), with patients with post-stroke depression (PSD), so as to provide a basis for the treatment and prevention of PSD for patients.

**Methods:**

The clinical laboratory data of 60 patients with PSD in The Second Affiliated Hospital of Qiqihar Medical University, Qiqihar, China from July 2016 to July 2017 and those of another 60 stroke patients without PSD admitted in the same period were analyzed retrospectively. The expression levels of inflammatory response factors in the two groups of patients and in PSD patients with different levels of depression were compared and analyzed via statistical methods. Multiple Logistic regression analysis was used to determine whether inflammatory response factors were independent risk factors for PSD patients.

**Results:**

The expression levels of IL-6, TNF-α and CRP in patients with PSD were significantly increased compared with those in patients without PSD, and the differences were statistically significant (*t*=6.429, *t*=6.355, *t*=5.792, *P*<0.001). The levels of IL-6, TNF-α and CRP had statistically significant differences between any two groups of mild, moderate and severe PSD patients (*P*<0.05). Results of multiple Logistic regression analysis revealed that the odds ratio (OR) values of inflammatory factors (IL-6, TNF-α and CRP) were 1.160, 1.099 and 1.248, respectively, and the corresponding *p* values were 0.020, 0.039 and 0.007 in patients of observation group, indicating the above three inflammatory response factors were independent risk factors for PSD.

**Conclusion:**

The clinic control on the expression levels of inflammatory response factors (IL-6, TNF-α and CRP) are extremely important for the treatment and prevention of PSD.

## Introduction

Stroke is currently the second leading cause of death in the world and is a common and frequent disease in neurological department (1). Post-stroke depression (PSD) is an affective disorder characterized by indifference, depression, lack of interest, anxiety and somnipathy and even suicide after stroke. The incidence rate of PSD is as high as 39%–52% (2). Its occurrence mechanism has become a research focus, mainly including immune factors, inflammatory response, nutritional cytokines and cytokines-neuro-endocrine-immune network, etc. (3). In recent years, inflammatory cytokines, such as interleukin (IL), tumor necrosis factor (TNF), C-reactive protein (CRP), have become a research focus.

In this study, we wanted to provide an effective solution for the diagnosis, treatment and prevention of PSD by collecting a large number of samples to statistically analyze the relationship between each indicator and PSD.

## Materials and Methods

### Clinical data

A total of 120 patients with stroke admitted in the Neurological Department of The Second Affiliated Hospital of Qiqihar Medical University, Qiqihar, China from July 2016 to July 2017 were selected. All the included patients were in line with the diagnostic criteria raised by the Fourth National Academic Meeting for Cerebrovascular Disease in 1995. The past medical history (hypertension history, diabetes history, smoking history, drinking history and stroke history) and general data (occupation and degree of education) of patients in the two groups were collected to conduct the statistical analysis.

The study was approved by the Ethics Committee of the Second Affiliated Hospital of Qiqihar Medical University and written informed consents were signed by the patients.

Inclusion criteria: Patients with cerebral hemorrhage or cerebral infarction at first onset, patients without consciousness disorder, severe language dysfunction, cognitive disorder and who can answer questions of clinician and fill in the form, patients with single acute responsible nidus and patients who did not conceal any personal emotions or disease condition and who and whose families signed the informed consent.

Exclusion criteria: Patients with severe neurological deficit symptoms [the National Institutes of Health Stroke Scale (NIHSS) score>24 points] or accompanied by consciousness disorder, patients with severe language disorders, such as sensory aphasia, severe motor aphasia or apraxia, failing to cooperate to complete the Hamilton Depression Scale (HAMD) score, patients suffering from mental disorders, such as schizophrenia or depression in the past, patients with severe cognitive disorder, patients with severe heart disease, lung disease, cancer, severe infections or organ dysfunction diseases, or patients with autoimmune diseases or long-term use of immunosuppressive agents.

### Grouping

PSD group (observation group): There were 60 patients with the HAMD score≥7 points in observation group, including 25 males and 35 females with an average age of (56.23±13.17) years old. According to the severity of depression and HAMD score, the PSD group was divided into mild (n=24, 8 points≤ HAMD score≤ 16 points), moderate (n=26, 17 points≤ HAMD score≤ 24 points) and severe group (n=10, HAMD score≥ 24 points).

Stroke without depression group (control group): A total of 60 stroke patients without depression admitted into The Second Affiliated Hospital of Qiqihar Medical University in the same period were selected as control group, including 28 males and 32 females with an average age of (54.38±11.20) years old.

### Test methods

IL-6, TNF-α and CRP detection methods: Antecubital venous blood were drawn from all subjects within 24 hours after admission, placed in the anticoagulant tube and sent to the laboratory in The Second Affiliated Hospital of Qiqihar Medical University. Blood samples were placed under low temperature and centrifuged to be tested. The main measurement method was ELISA.

All the included patients were assessed according to the HAMD under the instructions of psychiatrists with rich experience in the hospital, and they were all in accordance with the diagnostic criteria of depression raised by the American Manual of Mental Disorders: HAMD≤7 points for no depression, 8 points≤ HAMD score≤ 16 points for mild depression, 17 points≤ HAMD score≤ 24 points for moderate depression, and HAMD≥ 24 points for severe depression.

### Statistical processing

The data of this study were all processed with Statistical Product and Service Solutions (SPSS) 22.0 (Chicago, IL, USA). The comparisons of enumeration data were based on the chi-square test statistical method, and the measurement data were described as mean ± standard deviation (x̄±s). The measurement data of the two groups were compared via independent-samples *t* test. Multiple Logistic regression analysis was applied to predict the risk factors. *P*<0.05 suggested that the difference was statistically significant.

## Results

### Comparisons of clinical data between the two groups of patients

There were no statistically significant differences between observation group and control group in gender, age, occupation, educational level, alcohol and tobacco history, history of hypertension, history of coronary heart disease and stroke type (*P*=0.581, 0.835, 1.00, 0.577, 0.831, 0.468, 0.707, 0.433, 0.680). The difference was statistically significant in the history of stroke complicated with diabetes (*P*=0.030, Table 1).

**Table 1:** Comparisons of age, gender, past medical history and general data of the two groups of patients (Each n=60)

***General data***	***Observation group n(%)***	***Control group n(%)***	***x^2^***	***P***
Gender (male)	25 (47.8)	28 (52.2)	0.304	0.581
Age (>45 yr old)	44 (49.4)	45 (50.6)	0.043	0.835
Occupation (with)	19 (50.0)	19 (50.0)	0.000	1.000
Degree of education (above high school)	26 (53.1)	23 (46.9)	0.310	0.577
Smoking history (Yes)	14 (48.3)	15 (51.7)	0.045	0.831
Drinking history (Yes)	18 (54.5)	15 (45.5)	0.527	0.468
Hypertension history (Yes)	38 (51.4)	36 (48.6)	0.141	0.707
Diabetes history (Yes)	24 (64.9)	13 (35.1)	4.728	0.030*
History of coronary heart disease (Yes)	13 (56.5)	10 (43.5)	0.616	0.433
Cerebral ischemic stroke (Yes)	43 (48.9)	45 (51.1)	0.170	0.680

Notes: There is no statistical significance between the two groups in terms of age, gender, smoking and drinking history, history of hypertension, history of coronary heart disease and stroke type (*P*>0.05). In observation group, the number of patients complicated with diabetes is significantly larger than that in control group, and the difference is statistically significant

* *P*<0.05

### Comparisons of changes in expression levels of serum inflammatory response factors in the patients of two groups

The differences between observation group and control group in the inflammatory response factors (IL-6, TNF-α and CRP) were statistically significant (*P*<0.05, Table 2).

**Table 2:** Comparisons of changes in expression levels of serum inflammatory response factors in the patients of two groups

***Variable***	***Observation group (n=60)***	***Control group (n=60)***	***t***	***P***
CRP (mg/L)	13.45±4.46	9.25±3.40	5.792	<0.001
IL-6 (pg/L)	16.83±6.51	10.50±3.81	6.492	<0.001
TNF-α (pg/L)	25.65±9.24	17.29±4.27	6.355	<0.001

Notes: The levels of IL-6, TNF-α and CRP in observation group and control group have statistically significant differences (*P*<0.05)

### Comparisons of expression levels of serum inflammatory response factors in patients with different depression degrees in observation group

The pairwise comparisons of IL-6, TNF-α and CRP in patients with different depression degrees in observation group showed statistically significant differences (*P*<0.05, Table 3).

**Table 3:** Comparisons of serum inflammatory response factor expression levels in patients with different depression degrees in observation group

***Variable***	***IL-6 (pg/L)***	***TNF-α (pg/L)***	***CRP (mg/L)***
Mild depression	11.79±3.84	16.87±4.93	10.19±2.46
Moderate depression	17.81±2.80	29.45±4.66	14.78±4.19
*t*	6.359	9.256	4.661
*P*	**>**0.001	<0.001	<0.001
Mild depression	11.79±3.84	16.87±4.93	10.19±2.46
Severe depression	27.11±4.33	37.56±3.41	17.81±3.39
*t*	10.200	12.054	7.399
*P*	<0.001	<0.001	<0.001
Moderate depression	17.81±2.80	29.45±4.66	14.78±4.19
Severe depression	27.11±4.33	37.56±3.41	17.81±3.39
*t*	7.625	4.985	2.236
*P*	<0.001	<0.001	0.037

Notes: The pairwise comparisons of inflammatory response factors in patients with different degrees of PSD show statistically significant differences. The higher the degree of depression is, the higher levels of inflammatory response factors will be

### Logistic regression analysis

In observation group, IL-6, TNF-α and CRP and other indicators were analyzed through multiple Logistic regression analysis (Table 4).

**Table 4:** Multiple Logistic analysis of indicators (IL-6, TNF-α and CRP) in observation group

***Variable***	***B***	***S. E***	***Wald***	***OR***	***P***	***95% CI***
IL-6 (pg/L)	0.128	0.058	4.915	1.160	0.020	1.015–1.273
TNF-α (pg/L)	0.097	0.041	5.642	1.099	0.039	1.017–1.194
CRP (mg/L)	0.144	0.070	4.229	1.248	0.007	1.007–1.326

Notes: Indicators, such as IL-6, TNF-α and CRP, are important risk factors for PSD patients

## Discussion

PSD is an affective disorder that occurs after stroke. The main clinical manifestations include depression, sleep disorder, sense of worthlessness, self-blame and even suicide. It mainly occurs within 1–2 months after stroke, whose pathogenesis is not yet fully understood at present. However, with the development of medicine and clinical understanding and research on PSD, more and more studies have proposed that PSD patients are accompanied by changes of inflammation cytokines (IL-6, TNF-α and CRP) and that the above inflammation cytokines participate in or mediate the formation of depression. This paper aims to further discuss the pathogenesis and treatment of PSD by retrospective comparative analysis of differences of the IL-6, TNF-α, CRP and other indicators in the stroke patients and PSD patients through statistical methods (4).

In this study, no significant correlations between PSD patients and stroke patients in terms of gender, age, education level, occupation, drinking history, smoking history, history of hypertension, history of coronary heart disease and stroke type were found. However, there was a certain correlation in history of diabetes between them. At present, there is no consensus that to which kind of complicated disease history PSD is related in China and overseas. It is mostly thought that the more the complications are, the higher the risk of PSD will be (5). The reason may be that the correlation between PSD and hyperglycemia is mainly related to insulin-like growth factor-1 (IGF-1), and blood glucose and immune factors can affect the level of IGF-1 in blood. Blood IGF-1 level in patients with diabetes and depression has significant changes, which is consistent with the result of another study (6). Serum IGF-1 level in PSD patients at different degrees of depression is different and the difference is statistically significant, which might be related to the fact that serum IGF-1 level could lead to PSD due to its impact on hypothalamus-pituitary-adrenal axis (7). However, its specific role in the pathogenesis of PSD still needs more discussion, so a certain correlation between diabetes and PSD may be related to the influence on the serum IGF-1 level.

We found that levels of IL-6, TNF-α and CRP and other inflammatory factors in observation group were significantly different from those in control group. The levels of IL 6, TNF-α and CRP in serum of patients with PSD at different levels were significantly different between any two groups in observation group. The injured brain tissues of PSD patients had degeneration, edema and necrosis and secreted a large number of inflammatory cytokines such as IL-6, TNF-α and CRP. The reasons why inflammatory cytokines can cause depression may be related to the following aspects:

- Inflammatory factors affect the concentration, quantity, function, transport and update of monoamine neurotransmitters in neuronal synapses by acting on monoamine neurotransmitters such as serotonin and norepinephrine, leading to the decrease in their quantity or function. Monoamine neurotransmitters play an important role in the maintenance of nervous system excitability, so the decrease in their number or function will result in the occurrence of depression.
- Inflammatory factors affect the limbic system-hypothalamo-pituitary-adrenal (HPA) axis: The HPA axis promotes cortisol secretion in the body by activating the hypothalamus, pituitary and adrenal glands. Cortisol in the normal range inhibits the release of inflammatory cytokines, whereas excessive cortisol can damage nerve cells through cytotoxicity. The release of a large number of inflammatory cytokines activates the HPA axis, resulting in its over-excitement that secretes large quantities of cortisol, leading to nerve cell damage.

Inflammatory factors may affect the generation of brain derived neurophic factor (BDNF) via some neural pathways. BDNF plays an important role in the structure and function of the brain regions which are responsible for the regulation of depression, and plays a crucial role in the proliferation and differentiation of neural cells and the reduction in its amount affects the neuro plasticity (8). Peripheral inflammatory response can lead to depression, the inflammatory factors (IL-6, TNF-α and CRP) are mainly synthesized by monocytes and produce inflammatory response and promote immune response (9–11). The above inflammatory factors lead to neurotransmitter secretion disorders in the body, thus resulting in depression (12). Inflammatory factors IL-6, TNF-α and CRP are important risk factors for PSD, suggesting that it is crucial to reduce the inflammatory response during the treatment for PSD patients (13).

## Conclusion

There is a clear correlation of serum inflammatory response factors with PSD patients. To some extent, the expression levels of the former reflect the severity of the disease in PSD patients and the active control of PSD patients with elevated levels of serum inflammatory response factors can reduce the incidence of PSD and improve life quality of patients. Therefore, the regulation of serum inflammatory response factors may become a new way to prevent and treat PSD.

## Ethical considerations

Ethical issues (Including plagiarism, informed consent, misconduct, data fabrication and/or falsification, double publication and/or submission, redundancy, etc.) have been completely observed by the authors.

## References

[1] Ayerbe L Ayis S Wolfe CD Rudd AG (2013). Natural history, predictors and outcomes of depression after stroke: systematic review and meta-analysis. Br J Psychiatry, 202: 14–21. 23284148 10.1192/bjp.bp.111.107664

[2] Pohjasvaara T Vataja R Leppävuori A (2001 ). Depression is an independent predictor of poor long-term functional outcome post-stroke. Eur J Neurol, 8:315–19. 11422427 10.1046/j.1468-1331.2001.00182.x

[3] Lambertsen KL Biber K Finsen B (2012). Inflammatory cytokines in experimental and human stroke. J Cereb Blood Flow Metab, 32: 1677–98. 22739623 10.1038/jcbfm.2012.88 PMC3434626

[4] Su JA Chou SY Tsai CS Hung TH (2012). Cytokine changes in the pathophysiology of poststroke depression. Gen Hosp Psychiatry, 34: 35–9. 22055333 10.1016/j.genhosppsych.2011.09.020

[5] Barker-Collo SL (2007 ). Depression and anxiety 3 months post stroke: prevalence and correlates. Arch Clin Neuropsychol, 22:519–31. 17462857 10.1016/j.acn.2007.03.002

[6] Casazza K Hanks LJ Alvarez JA (2010). Role of various cytokines and growth factors in pubertal development. Med Sport Sci, 55:14–31. 20956857 10.1159/000321969

[7] Raha D Nehar S Paswan B (2007). IGF-I enhances cortisol secretion from guinea-pig adrenal gland: in vivo and in vitro study. Int J Mol Med, 20: 91–5. 17549394

[8] Curley AA Eggan SM Lazarus MS Huang ZJ Volk DW Lewis DA (2013). Role of glutamic acid decarboxylase 67 in regulating cortical parvalbumin and GABA membrane transporter 1 expression: Implications for schizophrenia. Neurobiol Dis, 50: 179–86. 23103418 10.1016/j.nbd.2012.10.018 PMC3534919

[9] Yasuno F Taguchi A Yamamoto A (2014). Microstrural in matter, regulatory T lymphocytes, and depressive symptoms after stroke. Psychogeriatrics, 14: 213–21. 25495082 10.1111/psyg.12084

[10] Lee SR Choi B Paul S (2015). Depressive-like behaviors in a rat model of chronic cerebral hypoperfusion. Transl Stroke Res, 6: 207–14. 25541087 10.1007/s12975-014-0385-3

[11] Loubinoux I Kronenberg G Endres M (2012). Post-stroke depression: mechanisms, translation and therapy. J Cell Mol Med, 16: 1961–9. 22348642 10.1111/j.1582-4934.2012.01555.x PMC3822966

[12] Anisman H (2009). Cascading effects of stressors and inflammatory immune system activation: implications for major depressive disorder. J Psychiatry Neurosci, 34: 4–20. 19125209 PMC2612083

[13] Overstreet DH Fredericks K Knapp D Breese G McMichael J (2010). Nerve Growth Factor (NGF) Has Novel Antidepressant-like Properties in Rats. Pharmacol Biochem Behav, 94: 553–60. 19945476 10.1016/j.pbb.2009.11.010 PMC3003426

